# Contemporary reflections on William Gull's case studies of anorexia nervosa, 150 years on

**DOI:** 10.1002/erv.3139

**Published:** 2024-10-14

**Authors:** Mark Mayall, Raja Sadhu, Brett McDermott

**Affiliations:** ^1^ James Cook University Townsville Townsville Queensland Australia; ^2^ Townsville University Hospital Townsville Queensland Australia; ^3^ Tasmanian Health Service Hobart Tasmania Australia

**Keywords:** anorexia nervosa, DSM‐5‐TR, eating disorders, ICD‐11, William Gull

## Abstract

**Objective:**

To analyse and compare the original four published anorexia nervosa (AN) case histories of William Gull with modern‐day approaches.

**Method:**

Case histories of the patients described by Gull were reviewed and placed in a tabulated format (which included demographics, clinical presentation, treatment, and prognosis) along with his general comments on AN, for easier comparison.

**Results:**

Many of the presenting features of AN are similar to cases seen in more modern times but lack weight or body image disturbances. The cases described by Gull can be categorised as AN under the Diagnostic and Statistical Manual‐Fifth Edition (DSM‐5) and the Text Revision (DSM‐5‐TR) however, they were excluded by the Diagnostic and Statistical Manual‐Fourth Edition (DSM‐IV) and the International Classification of Diseases‐10th Revision (ICD‐10) criteria. Reference to Gull's work might have avoided the necessary change in diagnostic criteria.

**Conclusions:**

150 years on, Gull's cases resemble presentations of AN without weight or body image issues and emphasise the heterogeneity of the diagnostic conceptualisation of AN in the modern era. Nutritional rehabilitation remains core to the treatment with other interventions supporting this goal while aetiology remains elusive.

## Key points

- Gull's cases resemble those cases of AN who do not have a fear of weight gain or body image disturbances, stressing the heterogeneity within the anorexia nervosa (AN) diagnosis.
- Gull's cases were not recognised as AN according to the diagnostic criteria of the diagnostic and statistical manual‐forth edition (DSM‐IV) and the international classification of diseases 10th version (ICD‐10); however, changed criteria in DSM‐5/DSM‐5‐TR and the international classification of diseases 11th revision (ICD‐11) allowed Gull's cases to be diagnosed as AN.
- As in Gull's cases, nutritional rehabilitation remains the core treatment of AN along with other supportive measures.

## 1 INTRODUCTION AND AIMS

It is over 150 years since Sir William Gull first briefly mentioned the eating disorder that he later named anorexia nervosa (AN). The mention is contained in a wide–ranging address to the British Medical Association in Oxford, in 1868, in a section devoted to abdominal diseases. Gull refers shortly and obliquely to a disorder of weight loss with no apparent gross peripheral organic disease. Gull initially referred to this condition as ‘Hysteric Apepsia’.

Gull was considered an eminent physician in his time and in 1871 was credited with saving the life of the Prince of Wales, becoming both a Baronet and one of Queen Victoria's Physicians–in–Ordinary.

In 1873, William Gull presented two cases (Miss A and Miss B) ‘as fair examples of the whole’ (subsequently published in 1874), and in an addendum, he added a further case (Miss C) referred by a Dr Anderson and quotes from three letters he received from him in April 1873 (Gull, 1874). In the published collection of his works (1896), a further case of AN (Miss K R) was added with a note from the editor; ‘The following case was Sir William Gull's last contribution to the study of clinical medicine’ (Gull, 1896, 1997, reprinted).

This article aims to analyse the cases described by William Gull in the context of modern‐day presentations/clinical features, the demographic features of patients with feeding and eating disorders, compare the changes in management and prognosis of these patients and also analyse the nosological status of the patients described by Gull according to recent classification systems.

## 2 METHODOLOGY

The cases described by William Gull were reviewed in terms of the language used by Gull himself. The criteria of feeding and eating disorder as described in recent classification systems were analysed to see which one fits the best and explains the cases as described by Gull.

## 3 RESULTS

Table 1 shows aspects of the four published cases along with some important general comments by Gull while Table 2 shows a summary comparison of the clinical features described in Gull's cases against information in the DSM‐5‐TR (American Psychiatric Association) and ICD‐11 (World Health Organisation).

**TABLE 1:** Clinical aspects of William Gull's cases of anorexia nervosa.

Descriptors	Case A	Case B	Case C	Case KR
Age	Approx. 17 yrs	Approx. 18 years	15 years 8 months	Approx. 14 years
Sex	Female	Female	Female	Female
Family history			‘No family history of disease beyond the fact that maternal grandmother had had peculiar nervous symptoms’.	‘The third child in the family of six; one of whom died in infancy. Father died, aged 68 of pneumonic phthisis. Mother living and in good health. Has a sister is the subject of various nervous symptoms and a nephew epileptic. With these exceptions there has been no other neurotic cases on either side of the family which is a large one’.
Aetiology and precipitants	‘No account could be given of the exciting cause’. ‘It will be observed that all the conditions in this case were negative, and may be explained by the anorexia which led to starvation, and a depression of the vital functions. In the stage of greatest emaciation one might have been pardoned for assuming that there was some organic lesion’.	‘Brought to me…as a case of latent tubercle’	‘I believe it to be essentially a failure of the powers of the gastric branches of the pneumogastric nerve. It differs from tuberculosis, though that state may subsequently arise, by the pulse, which I found to be 64, by the breathing, 16, the cleanness of the tongue, &c. In fact, the disease will be most correctly interpreted if it is remembered that no symptom more positive than emaciation is presented in and throughout its course’.	‘An illustration of most of these cases, perversions of the “ego” being the cause and determining the course of the malady’.
General comments: ‘The want of appetite is, I believe due to morbid mental state, I have not observed in these cases any gastric disorder to which the want of appetite could be referred. I believe, there, that its origin is central and not peripheral…. We might call the state hysterical without committing ourselves to the etymological value of the word, or maintaining that the subjects of it have common symptoms of hysteria. I prefer, however, the more general term “nervosa,” since the disease occurs in males as well as females, and is probably rather central than peripheral. The importance of discriminating such cases in practice is obvious; otherwise prognosis will be erroneous, and treatment misdirected’. ‘The subjects of this affection [AN] are mostly of the female sex, and chiefly between ages of 16 and 23. I Have occasionally seen it in males at the same age’. ‘To illustrate the disease I may give the details of two cases, [case A and B] as fair examples of the whole’.				
Physical examination/ Appearance/ Symptoms/ Special investigations	‘Her emaciation was very great. Amenorrhoea for nearly a year. No cough. No vomiting nor diarrhoea. Slight constipation. Complete anorexia for animal food, and complete anorexia for everything else. Abdomen shrunk and flat. The condition was one of simple starvation’. ‘The patient complained of no pain, but was restless and active. This was in fact a striking expression of the nervous state for it seemed hardly possible that a body so wasted could undergo the exertion which seemed agreeable’. ‘Occasionally for a day or two the appetite was voracious, but this was very rare and exceptional’. ‘was restless and active’. ‘It will be noticeable that as she recovered she had a much younger look… the photographs taken when she was 17, give her appearance of being near thirty’. ‘Pulse 56 and 60’ Weight; initial (17th January 1866) 82 lbs [37.2 kg] Premorbid weight 115 lbs [52.2 kg] ‘it was stated she had lost 33 lbs’ End weight (Mar 1868) 128 lbs [58.1 kg] Height initial‐ 5 ft 5ins [1.65 m] BMI initial 13.7 BMI end 21.4 (unknown height, assume initial height)’	‘Pulse 50, resp 16. Physical examination of the chest and abdomen discovered nothing abnormal. Notwithstanding the great emaciation and apparent weakness, there was a peculiar restlessness, difficult I was informed, to control. The mother added, “she is never tired.” Amenorrhoea since Christmas 1866.’ Pulse 56, resp, 12; January 1868, pulse 54. But little change occurred in the case until 1872, when the respirations became 18–20, pulse 60.	‘The clinical history was that she had been ailing for a year and had become extremely emaciated. The catamenia had never appeared. Pulse 64, resp. 16. Very sleepless for 6 months past. All the viscera healthy. Urine normal. Lower extremities oedematous’ ‘Great restlessness’	‘The patient, who was a plump healthy girl until the beginning of last year (1887), began early in Feb, without apparent cause, to evince a repugnance to food, and soon afterwards declined to take any whatever except half a cup of tea or coffee’. ‘She was then extremely emaciated and persisted in walking through the streets to my house, though an object of remark to the passers–by’. ‘As part of the pathological history, it is curious to note, as I did in my first paper, the persistent wish to be on the move, though the emaciation was so great and the nutritive functions at an extreme ebb’. ‘She was then extremely emaciated’ ‘Examination showed no organic disease’ ‘Resp 12–14; pulse 46, temp 97. Weight 4st 7 lbs; [28.6 kg] height 5 ft 4in.’ [1.63 m] [BMI 10.75] ‘Extremities blue and cold’.
General comments: ‘My experience supplies one instance at least of a fatal termination to this malady. When the emaciation is at the extremest, oedema may supervene in the lower extremities, the patient may become sleepless, the pulse quick, and death approached by the symptoms of feeble febrile reaction’. ‘Death apparently followed from the starvation alone’. This is the clinical point to be borne in mind, and is, I believe, the proper guide to treatment. ‘It being often necessary to supply external heat as well as food to patients’.				
Mental health symptoms	‘There was some peevishness of temper, and a feeling of jealousy’.		‘Mind weakened. Temper obstinate. Great restlessness’. ‘The great difficulty was to keep her quiet, and to make her eat and drink. Every–step had to be fought. She was most loquacious and obstinate, anxious to overdo herself bodily and mentally. (Dr A)’	
Treatment	‘The diet was also varied, but without effect upon the appetite’ ‘Various remedies were prescribed—the preparations of cinchona, the bichloride of mercury syrup of the iodide of iron, syrup of phosphate of iron, citrate of quinine and iron, &c,‐ but no perceptible effect followed their administration’	‘The medical treatment probably need not be considered as contributing much to the recovery. It consisted, as in the former case, of various so–called tonics and a nourishing diet’.	‘I would advise warm clothing, and some form of nourishing food every 2 hours, as milk, cream, soup, eggs, fish, chicken. I Must only urge the necessity of nourishment in some form. With the nourishment I would conjoin a dessert–spoonful of brandy every two or 3 hours. Whilst the present state of weakness continues, fatigue must be limited, and if the exhaustion increases beyond its present degree the patient should for a time be kept in a warm bed. I Do not at present prescribe medicines, because the nursing and the food are more important than anything else’.	‘A nurse was obtained from guys [hospital] and light food ordered every few hours’.
General Comments: ‘The inclination of the patient must be in no way consulted’. In the earlier and less severe stages it is not unusual for the medical attendant to say, in reply to the anxious solicitude of the parents, “let her do as she likes. Don't force food”. ‘Formerly I thought such advice admissible and proper, but larger experience has shown plainly the danger of allowing the starvation process to go on’. ‘The restless activity referred to is also to be controlled, but this is often difficult’. ‘By warmth and steady supplies of food and stimulants, the strength may be gradually resuscitated, and recovery completed’. ‘it being often necessary to supply external heat as well as food to patients. The best means of applying heat is to place an India–rubber tube, having a diameter of 2 inches and a length of 3 or 4 feet, filled with hot water, along the spine of the patient as suggested by Dr Newington of Ticehurst’. ‘I have remarked that these wilful patients are often allowed to drift in their own way into a state of extreme exhaustion, when it might have been prevented by placing them under different moral conditions’. ‘The treatment required is obviously that which is fitted for persons of unsound mind. The patients should be fed at regular intervals, and surrounded by persons who would have moral control over them; relations and friends being generally the worst attendants’.				
Length of time in clinical setting	17th January 1866–March 1868 [27 months]	8th October 1868–1872. ‘After that date the recovery was progressive, and at length complete.’ [at least 40 months]	‘was sent to me in Apr 1873. The clinical history was that she had been ailing for a year’ [Apr 1872] ‘has now made a complete recovery, and is getting plump and rosy as of yore…’ (Dr A‐ Apr 1874) [Approximately 2 years]	Feb–Jul 1887 [6 months]
Prognosis and outcome	‘the satisfactory course of the case to entire recovery and by the continuance of good health’.	‘After that date the recovery was progressive, and at length complete.’	‘has now made a complete recovery, and is getting plump and rosy as of yore…’ (Dr A)	‘Jul 27th the mother wrote, “K—is nearly well. I Have no trouble now about her eating. Nurse has been away 3 weeks.”
General comments: ‘My experience supplies one instance at least of a fatal termination to this malady’. ‘As regards prognosis, none of these cases, however exhausted, are really hopeless whilst life exists; and, for the most part, the prognosis may be considered favourable’.				

**TABLE 2:** Comparison of clinical features of anorexia nervosa between Gull's cases and DSM‐5‐TR and ICD‐11 information.

Clinical descriptors	Gull's cases (see Table 1 for details)	DSM‐5‐TR (APA, 2022)	ICD‐11 (WHO, 2024)
Age	‘chiefly between ages of 16 and twenty–three’	‘It rarely begins before puberty or after age 40’	Often between 10 and 24 years
Sex	‘mostly of the female sex’	Lifetime prevalence 0%–0.08% in women to 0%–0.01% in men.	‘up to 10 times more commonly diagnosed among females’
Family history	Some non‐specific ‘nervous symptoms’	‘There is an increased risk for anorexia nervosa and for other eating and psychiatric disorders among biological relatives’	Not covered
Aetiology and precipitants	‘No account could be given of the exciting cause’.	Aetiology not specified. ‘Often associated with a stressful life event’	Aetiology not specified. ‘typically following a stressful life event’.
Physical examination/ Appearance/ Symptoms/ Special investigations	Weight loss and associated features ‘Restlessness’ (No organic cause found)	Weight loss and associated features ‘Some individuals …show excessive levels of physical activity’	Weight loss and associated features ‘Excessive exercise, motor hyperactivity’
Mental health symptoms	No fear of fatness or body image issues reported Some non specific symptoms	Can be associated with fear of weight gain/becoming fat and body image disturbance (see Table 3 for details) Co–morbidities: OCD, mood and anxiety disorders, substance use disorders.	Typically associated with: • Extreme fear of weight gain • Excessive preoccupation with body weight or shape An explicitly stated fear of weight gain is not an absolute requirement for the diagnosis. Boundaries with other disorders and conditions: Other eating disorders Schizophrenia and primary psychotic disorders OCD/Body dysmorphic disorder
Treatment	Weight restoration/‘moral control’	Not covered	Not covered
Length of time in clinical setting	6 to at least 40 months	‘Most individuals with AN experience remission within 5 years’	Most have ‘remission within 5 years’
Prognosis and outcome	‘prognosis may be considered favourable’ ‘one instance at least of a fatal termination’	‘crude mortality rate…approximately 5% per decade’	‘The prognosis for adolescents…is better than the prognosis for adults’ ‘associated with premature death often due to medical complications of starvation or to suicide’

### 3.1 Clinical presentations of William Gull's cases of AN

In age, Gull's published cases ranged from 14 to 18 years (the majority in his practice between 16 and 23), and all were female (though he had seen the disorder in males).

There are several woodcuts representative of original photographs which are of interest. The illustrations show the patients in what appears to be quality clothing with ornamentation and jewellery (and elaborate hairstyling) which along with Gull's eminent position at the time and the lack of an established public health service strongly suggests that these patients were from a high socioeconomic position and that Gull was seeing them in his private practice.

As is common with many presentations of AN, Gull's cases all presented with an already significant degree of weight loss. Gull mentioned amenorrhoea and in one case infrequent binging episodes as symptoms in his patients. Tuberculosis, which often presented with weight loss, was considered a differential diagnosis in these cases, however, there was no supporting evidence for tuberculosis in the patients presented. Any physical abnormalities such as low pulse rate seemed related to nutritional compromise with Gull concluding that anorexia had led directly to starvation. As regards aetiology, no direct cause could be found, and Gull concluded that the anorexia was ‘due to morbid mental state’. In summary, his view was that it was the significant weight loss and lack of discernible organic pathology which marked out the diagnosis of AN.

Gull mentions some background factors in two of his patients like the loss of a sibling, a potentially significant death, nervous symptoms, epilepsy in the family members of one of them and nervous symptoms in a family member of the other patient. Restlessness and significant activity, even in states of severe weight loss, were noted in all the cases and seemed to have made a significant clinical impression. Where mental health symptoms were present, they were primarily the reluctance to eat and subsequent loss of weight, though some temperamental problems were noted in two of his patients.

### 3.2 Treatment and prognoses of William Gull's cases of AN

As regards the treatment that Gull outlines, it consisted of the following: an approach that recognised that this was a disorder over which the patient had little control. He also emphasised that the wishes of the patient should not be taken into consideration and that relations and friends might be countertherapeutic.

The main direct treatment Gull emphasised was nutritional rehabilitation. However, his advice ‘Food should be administered at intervals varying inversely with the exhaustion and emaciation’ suggests a knowledge of the dangers of refeeding syndrome. This should not be a surprise for this danger had been known since classical times. Gull tried several common medical remedies at use at that time but concluded that they were ineffective. In one of his patients a nurse from Guy's Hospital, London, where Gull practised, was procured to assist with the treatment. The length of time in the treatment of the four cases, as far as can be discerned, was between 6 months and approximately 40 months. As for prognosis of the cases he was seeing in his clinical practice, he was generally positive though adding he had seen one fatality.

## 4 DISCUSSION

### 4.1 150 years on–The current situation ‐ What have we learnt?

#### 4.1.1 Clinical presentations: Comparison with Gull's cases

In many ways, the cases discussed by Gull are quite familiar to the modern psychiatrist and his work continues to be influential (Madden, 2004). Demographically, Gull's description of a preponderance of young females with occasional males fits with more contemporary research (Lock, 2019; Petkova et al., 2019). Familiar also is the late presentation to medical services with significant emaciation, amenorrhoea, and activity out of keeping with the degree of emaciation. Others reviewing Gull's cases speculated that this activity might not, as commonly assumed, be just to do with weight control and could be something much more fundamental to AN (Casper, 2016).

Gull mentioned no clear, consistent, or specific precipitating factors, though he was aware of some of the psychological aspects, mentioning as he did some background factors which might be considered as nonspecific factors and stressors. He was also familiar (in discussing the etymology of AN) with the then–contemporary view of ‘hysteria’.

#### 4.1.2 Gull's cases and the fear of weight gain–The possibilities

Gull did not describe the fear of gaining weight or disturbed body image. This might reflect the fact that the symptoms were not reported/absent by the patients or differing 19th‐century sociocultural influences (Habermas, 2015). However, it might also underscore the inherent symptomatic heterogeneity contained in the DSM‐5‐TR AN definition (e.g., fat‐phobic vs. non‐fat phobic) (Dalle Grave et al., 2008; Korn et al., 2020; Lee et al., 1993, 2012; Mustelin et al., 2016). Gull appears to have had a keen interest in mental health as in 1868 he wrote a paper on hypochondriasis and later described adult hypothyroidism commenting on its effect on the mind.

Writing around the same time as Gull an eminent French physician, Lasegue also described the syndrome of anorexia (anorexia hysterica) but only in females perhaps reflecting the strong French historical tradition associated with the concept of hysteria. Lasegue likewise did not describe any cases which might be considered ‘fat phobic’. The fact that these two extremely experienced clinicians did not mention any of these symptoms makes it far less likely that they missed any ‘fat phobic’ symptoms. Weight phobia was described in patients by later contemporaries (Habermas, 2015) and modern research supports the idea that both Gull and Lasegue may have seen patient groups which some have labelled ‘non‐fat phobic’ that is, those low weight eating disorders who deny fear of weight gain (Dalle Grave et al., 2008; Lee et al., 1993, 2012). In one recent study up to a third of hospitalised patients with AN failed to score significantly on standardised eating disorder questionnaires, one possibility being that ‘the existing measures do not capture the full range of AN presentations, such as non–fat phobic AN’ (Vanzhula et al., 2024, p. 796). This raises some very important questions about AN itself. Control of feeding is a highly complex system integrating peripheral neurohormonal, substantial vagal and other peripheral neuronal connections and a complex array of central systems (Andermann & Lowell, 2017; Stover et al., 2023).

Control of eating behaviour in patients with eating disorder has been linked to their personality styles where patients with AN and a typical AN have been found to show overcontrol (characterised by rigidity, perfectionism, and inhibition of feelings) in their personality as compared to other eating disorder like bulimia bervosa (Isaksson et al., 2021). However other authors prefer the term “coping” rather than “control” as a central factor as they argue eating disorder could be a result of life events or environmental factors (Branley‐Bell et al., 2023).

AN has been conceptualised recently as having multiple possible aetiologies (Gorwood et al., 2016). Could the heterogeneity of symptoms reflect differing processes of pathophysiology, that is, are different forms of anorexia nervosa (perhaps with differing aetiologies) hidden in our current diagnostic category?

Does the ‘non‐fat phobic’ variety apparently seen by Gull represent the true basic form of the disorder or is it the ‘forme fruste’, that is a partial or attenuated form of the ‘fat‐phobic’ disorder? The apparent low levels of ‘fat–phobic’ variety found in non‐western samples in the past might support the former view. In that case, could the ‘fat phobic’ symptom cluster be a culturally determined additional perpetuating factor for an underlying basic form of anorexia perhaps with more biological origins? Might the explanations for weight loss given by some sufferers of AN be attempted cortical rationales for unconscious subcortical pathology? Gull's cases therefore continue to raise important questions about how this disorder should be understood while Gull's observations on the restless activity (and subsequent activity–based animal models) along with Gull's suggestion of the use of heat in treatment suggest other avenues of enquiry and show the continuing influence of Gull's work even now (Carrera & Gutiérrez, 2018; Moncrieff‐Boyd, 2016; Spadini et al., 2021).

#### 4.1.3 Nosology—The status of Gull's cases—The journey through the time

The DSM‐IV criteria, which largely followed the diagnostic and statistical manual third edition (DSM‐III, published 1980) and the diagnostic and statistical manual third edition‐ revised (DSM‐III‐R, published 1987), excluded cases like those described by Gull, resulting in a substantial number of eating disorders being classified as eating disorder not otherwise specified (EDNOS) rather than AN (Fairburn & Bohn, 2005). By contrast the international classification of diseases version‐ninth revision (ICD‐9, agreed 1975, published 1977) criteria mentioned neither fear of fatness nor body image disturbance, the main criteria being ‘persistent active refusal to eat and marked loss of weight’.

In the DSM‐5‐TR one might cluster at least four possible types of AN; see Table 3 where the ‘B’ and ‘C’ criteria have been separated into B(i)/B(ii) and C(i)/C(ii). All the four diagnostic clusters require the DSM‐5‐TR ‘A’ criteria to be met, however, having met this criterion, as the table suggests, it is possible to have (1) A fear of weight gain/fatness B(i) and disturbance of body image (weight/shape) C(i); (2) Fear of weight gain/fatness B(i) and a lack of recognition of the seriousness of low weight C(ii); (3) Behaviour that interferes with weight gain B(ii) and lack of recognition of seriousness of low weight and C(ii); (4) Behaviour that interferes with weight gain B(ii) and disturbance of body image (weight/shape) C(i). All four types of theoretical diagnostic clusters can also have either Restricting–type or a Binge–eating/purging type leading to eight possible clusters of symptoms admitted under the AN diagnosis.

**TABLE 3:** Illustration of symptom heterogeneity within current DSM‐5‐TR anorexia nervosa diagnosis.

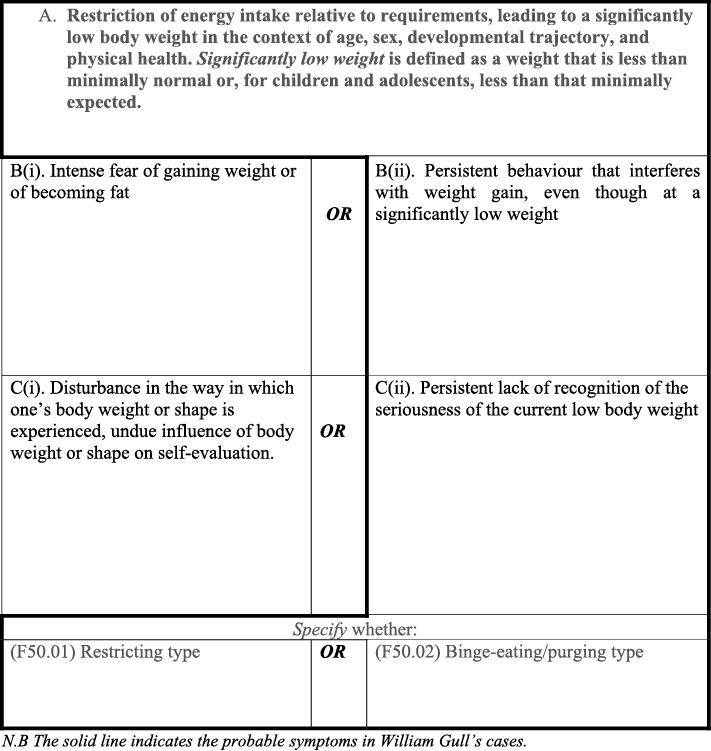

If the distinction made in the DSM‐5‐TR criteria between weight and shape in self‐evaluation and the separation of fear of gaining weight and fear of becoming fat is insisted upon, then further symptom clusters could emerge. Gull seems to be describing those features surrounded by a bold border in Table 3 and specifically not mentioning the fear of fatness—‘fat phobic’ AN or body image disturbance (labelled B(i) and C(i) in Table 3). Although the relatively recent diagnosis of avoidant/restrictive food intake disorder (ARFID) cannot be entirely ruled out for the four cases described by Gull, the age range alone does not seem promising, neither are commonly associated features of ARFID mentioned by Gull.

The ICD‐11 definition of AN broadly follows the same pattern as that of DSM‐5‐TR; Though ICD‐10 excluded cases like those described by Gull, according to ICD‐11 ‘An explicitly stated fear of weight gain is not an absolute requirement for the diagnosis of anorexia nervosa’.

It is interesting to consider if the differences in the historical DSM and the ICD criteria reflect different frequencies of AN presentations in the US or perhaps the result of particular socio‐cultural influences as suggested by others (Bemporad, 1997; Dell’Osso et al., 2016; Lee et al., 1993). It is possible that a historical perspective, which was cognisant of Gull's and his contemporary's work, might have avoided the necessity of the diagnostic criteria change from DSM‐IV to DSM‐5.

#### 4.1.4 Gull's management of anorexia nervosa: The progress we have made

The treatment approaches that Gull advised are also familiar, with weight restoration as the prime treatment offered and an acknowledgement that medications were generally unable to alter the primary pathology, a situation that has continued. Gull mentioned some important general principles regarding the approach to these patients, such as a framework that challenged the eating disorder within the young person, ‘The inclination of the patient must be in no way consulted’ and emphasised the need for supervision of the nutritional rehabilitation which appears a necessary but often not a sufficient treatment. Gull's admonition of the family and friends as poor attendants is also familiar. Gull's contemporary, Charcot's observation, that ‘Prayers, entreaties, violence, are unable to overcome their resistance’ (Silverman, 1997) reflects the inadequacy of behavioural or psychological interventions, patients with AN cannot generally be ‘talked’ out of their condition. In more modern times differing treatment strategies have been developed including family based treatment (FBT) which is a manualized treatment for Anorexia with the first phase being weight gain (Lock, 2019) and the active involvement of the family and patient, drawing on the experience of family dynamics (especially helped by the insights from family therapy) along with targeted psychological therapies like CBT. In adults, the Maudsley Anorexia Nervosa Treatment for Adults model has also been shown to have efficacy with nutrition a core component (Startup et al., 2021) Nursing support was also mentioned by Gull and sometimes the family and wider system seem to become the equivalent of the nurse procured from Guy's hospital to help in one of Gull's cases. Today good working relationships with paediatrics and nursing can often be essential, especially in the early stages of treatment. However, fundamentally the supportive treatments as in Gull's time remain focused on restoration and maintenance of weight gain. The more recently developed intervention for ED, Specialist Supportive Clinical Management, for instance, takes a similar pragmatic approach of clinical management along with supportive psychotherapy (Kiely et al., 2022). Unfortunately, more modern interventions can in no way be seen as ‘magic bullets’ and there remains, in the 21st century, still no definitive or curative treatment for AN. Also familiar from Gull's cases is the often‐prolonged clinical course sometimes over years, even with eventual recovery. Gull mentioned a generally favourable prognosis of his cases (though he may have seen, as discussed, a selected non‐fat phobic group) while today AN continues with a moderate outcome for adolescent anorexia with the proviso of significant morbidity during its often‐chronic course and with the threat of future mortality if treatment is unaddressed (Lock, 2019).

#### 4.1.5 Gull's cases‐The search for answers continue

As to the causation of AN, this appears to be complex and multifactorial with biological, psychological and socio‐cultural factors all appearing to play a part. Although many theories abound as to possible aetiological processes (Gorwood et al., 2016; Lock, 2019) like Gull, modern researchers are still searching for answers. However, unlike in the 19th‐century there are many more scientific tools available today to analyse the myriad of potential aetiological models. Our understanding of for instance genetics (Paolacci et al., 2020) and the neurobiology of feeding is certainly becoming more sophisticated (Andermann & Lowell, 2017). General advances in our understanding of both medical and mental disorders can only help in the search for answers to a condition that remains in many ways as puzzling and challenging a century and a half from Gull's original publication on AN.

## CONFLICT OF INTEREST STATEMENT

The authors declare that no funds, grants, or other support were received during the preparation of this manuscript. There are no conflicts of interest.

## Data Availability

The article is based on an analysis of clinical cases from the 19th century which are in the public domain.
